# A postoperative in situ drug delivery system based on biphasic drug-release and “Three-in-One” Effect of curcumin to inhibit the recurrence of glioma

**DOI:** 10.1016/j.ijpx.2025.100418

**Published:** 2025-10-12

**Authors:** Qiong Liang, Jiangjian Liu, Yanhang Zhuo, Sunhui Chen

**Affiliations:** Shengli Clinical Medical College of Fujian Medical University, Department of Pharmacy, Fuzhou University Affiliated Provincial Hospital, Fuzhou 350001, China

**Keywords:** Postoperative glioma recurrence, Postoperative in situ drug delivery system (PIDDS), Curcumin sensitization, Biphasic drug release, ROS-sensitive gel

## Abstract

The standard treatment for glioma is surgical resection of the tumor followed by postoperative temozolomide-based radio chemotherapy. However, the survival rate remains poor. This study, aiming to address the challenge of postoperative glioma recurrence, developed a reactive oxygen species-sensitive and thermo-sensitive gel to form biphasic drug-release postoperative in situ drug delivery system (PIDDS). This system simultaneously carried free temozolomide, free curcumin, and drug-loaded PLGA nanoparticles. Through a “rapid release + sustained release” biphasic drug-release mode and the “three-in-one” effect of curcumin, it enhanced the chemo sensitization of temozolomide, inhibited the glioma stem cells, and regulated the postoperative recurrence microenvironment, to achieve synergistic tumor recurrence inhibition. In vitro and in vivo experiments have shown this dual-sensitive gel PIDDS had a cumulative drug-release rate of over 88 % within 30 days, significantly extending the median survival time of rats to 57 days, 3 times higher than that of control group, while reducing systemic toxicity. The study has confirmed the PIDDS worked by disrupting DNA repair, inhibiting JAK-STAT stemness pathway, and reprogramming metabolic microenvironment, thus providing a new strategy for precise postoperative treatment of glioma.

## Highlights

Our study (A Postoperative In in Situ Drug Delivery System Based on Biphasic Drug-Release and “Three-in-One” Effect of Curcumin to Inhibit the Recurrence of Glioma, ID: IJPHARMX-D-25-00212R1) has 3 highlights:

• **1. A biphasic drug-release postoperative in situ gel system** was developed, integrating rapid and sustained release.
• **2. Curcumin exerts a “three-in-one” synergism** by enhancing chemosensitivity, inhibiting GSCs, and reprogramming TME.
• **3. The ROS- and thermo-sensitive gel system** enables localized, responsive drug delivery within the resection cavity.

## Graphical abstract

This study, aiming to address the challenge of postoperative glioma recurrence, developed a reactive oxygen species-sensitive and thermo-sensitive gel to form biphasic drug-release postoperative in situ drug delivery system, through a “rapid release + sustained release” biphasic drug-release mode and the “three-in-one” effect of curcumin, to achieve synergistic tumor recurrence inhibition.

**Figure:**
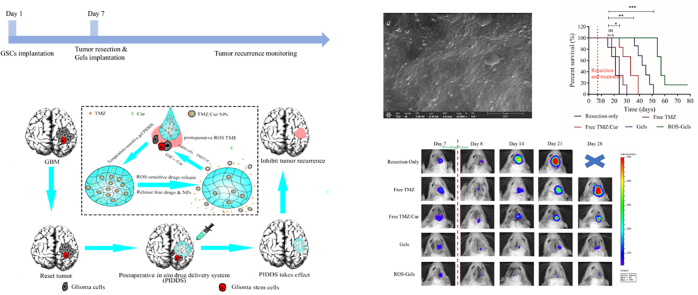

## 1 Introduction

Gliomas are the most common primary intracranial tumors, among which glioblastoma multiforme (GBM) is the most invasive and most malignant grade IV glioma (Hulou and Chiocca, 2015). The current clinical standard treatment involves maximal safe resection of the primary tumor, followed by radiotherapy and adjuvant chemotherapy with temozolomide (TMZ) (Fisher and Adamson, 2021). Surgical resection of glioma can alleviate clinical symptoms and provide a definitive pathological diagnosis. It is the first and most crucial step in the treatment of glioma. However, due to the infiltrative growth of GBM, complete resection of tumor tissue is difficult (Hervey-Jumper and Berger, 2019). The presence of the blood-brain barrier (BBB) also greatly limits the selection of systemic chemotherapeutic agents. Although the first-line chemo-drug TMZ can penetrate the BBB, its concentration in brain is only 20–30 % of the plasma concentration (Eudocia et al., 2025). Moreover, tumor cells are prone to developing resistance to TMZ. These factors have led to the still unsatisfactory therapeutic outcome for GBM, with a median survival of less than 15 months and a 5-year survival rate of less than 5 % among patients. On the other hand, current research mostly focuses on penetrating the BBB and targeting tumor cells to achieve the desired therapeutic effects. This treatment strategy is inconsistent with the clinical standard treatment protocol, and patients must continually endure the presence of primary GBM. Additionally, the drug concentration in the tumor region is too low due to systemic administration, resulting in poor outcomes in relevant clinical trials (Qiao et al., 2022). In contrast, postoperative in situ drug delivery system (PIDDS) can directly deliver drugs to the tumor resection cavity through nanomaterials or formulation technologies, completely bypassing the BBB and greatly expanding the possibilities for treatment. Therefore, the development of a novel PIDDS for inhibiting postoperative recurrence of GBM holds significant theoretical and clinical application value.

PIDDS refers to a drug delivery strategy that involves directly implanting a drug delivery system into the resection cavity immediately after surgery. When applied to the treatment of postoperative recurrence of GBM, PIDDS offers the following advantages: 1) Applicability for postoperative recurrence prevention of brain gliomas. If a tumor is prone to distant metastasis, tumor cells may have already spread to other tissues and organs by the time of surgery. Since PIDDS delivers drugs within the surgical cavity, its ability to track and kill tumor cells that have metastasized is limited. However, the unique anatomical structure of the brain means that over 90 % of recurrent brain tumors occur within a 2 cm range from the primary site, with minimal likelihood of metastasis to organs outside the central nervous system (Kambe et al., 2024). This makes the in situ treatment advantage of PIDDS in preventing postoperative recurrence of brain tumors fully realized. 2) Bypassing the BBB for dose reduction and toxicity minimization. Most drugs have poor permeability across the BBB, and only 0.1–1.0 % of the initially injected dose of systemic chemotherapeutic agents can enter the brain, which is insufficient to effectively kill brain tumor cells (Qiao et al., 2022). PIDDS, modified with suitable low-toxicity or non-toxic carrier materials or nanotechnology formulations, can significantly reduce the spatial distance between the drug and the tumor target site. Even with a reduced dose, the drug concentration at the target site can be effectively increased. Meanwhile, the systemic toxic side effects caused by the reduced dose of drugs are also relatively decreased. 3) Direct action on the postoperative tumor microenvironment that promotes recurrence. The resection cavity contains a complex postoperative tumor microenvironment represented by residual tumor cells, consisting of immune cells and their secreted factors, astrocytes, extracellular matrix, and blood vessels. This microenvironment promotes the recurrence of residual tumors. PIDDS can directly modulate the microenvironment in the resection cavity in situ, eliminating factors that favor tumor recurrence. 4) Integration with surgery to fill the treatment gap. In clinical practice, patients undergo an assessment period of about one month before starting radio chemotherapy. This treatment gap is a critical period for tumor recurrence (Johnson, 2000). PIDDS can provide immediate treatment tailored to the characteristics of GBM and the postoperative microenvironment, achieving the best possible effect in inhibiting recurrence. 5) Facilitation of synergistic effects of combination therapy. PIDDS makes it easier to achieve synergistic effects from combination drug therapies.

Currently, the only available postoperative PIDDS for glioma is the Gliadel wafer, which has been approved by the U.S. FDA. It uses a biodegradable polymer, polyanhydride-20, as the release matrix and carmustine as the active ingredient. However, its therapeutic efficacy is limited (Shapira-Furman et al., 2019), mainly due to 3 reasons: 1) The stubborn presence of glioma stem cells (GSCs). GSCs possess the ability for self-renewal and heterogeneous differentiation (Ma et al., 2018). They play a crucial role in glioma recurrence and resistance to radio chemotherapy, acting as the hard-to-eradicate “seeds” of tumor recurrence. 2) The postoperative tumor microenvironment promotes recurrence. The Gliadel wafer cannot inhibit this postoperative tumor microenvironment and may even cause complications related to infection and wound healing (Paul et al., 2003), which are even more detrimental to inhibiting the recurrence of brain tumors. 3) Difficulty in sustained drug release. The continuous release of carmustine from the Gliadel wafer lasts only 3–4 days. Subsequently, a “sink effect” occurs, causing the drug to diffuse into the systemic circulation (Attenello et al., 2008). This leads to insufficient drug concentration in the residual tumor area of the resection cavity, making it difficult to sustainably inhibit tumor recurrence.

Curcumin (Cur) is derived from the roots of plants in the ginger family. It has long been used as a spice and dye, and it is also employed in the treatment of diseases such as cancer, skin injuries, and tumors. Cur can inhibit the proliferation and metabolism of cancer stem cells, thereby reducing their stemness. This anti-tumor effect is achieved by suppressing various oncogenic molecules, including transcription factors, growth factors, tumor cytokines, and protein kinases (Shabaninejad et al., 2020). More importantly, recent studies have suggested that Cur can enhance the anti-tumor activity of chemotherapeutic drugs such as oxaliplatin, doxorubicin, erlotinib, and TMZ (Chao-Jan Liu et al., 2024). Therefore, the combination of TMZ and Cur can exert a stronger inhibitory effect on tumor cells. Additionally, the residual tumor microenvironment within the resection cavity after GBM surgery can promote the rapid recurrence of residual tumor cells. However, Cur can help suppress this tumor-promoting response (Kim et al., 2022).

To develop and evaluate a novel PIDDS that utilizes a biphasic release strategy and the multi-functional properties of curcumin to simultaneously target residual glioma cells, GSCs, and the recurrence-promoting microenvironment, thereby achieving potent and long-lasting inhibition of glioma recurrence.

**Scheme 1:**
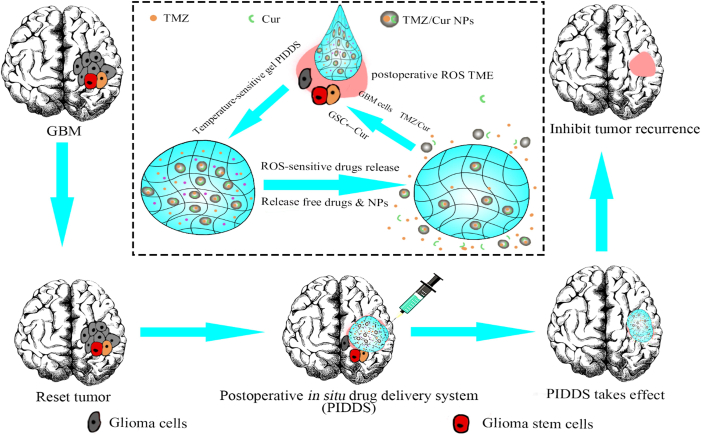
The thermo-sensitive postoperative in situ drug delivery system sensitively releases drugs in the ROS microenvironment to inhibit the recurrence of GBM.

Based on our previous research (Chen et al., 2022) and network pharmacology, we anticipate that incorporating Cur into the PIDDS will achieve a “three-in-one” effect: 1) enhancing the chemosensitivity of tumor cells to TMZ and synergistically inhibiting tumor cells; 2) suppressing the stemness of GSCs; and 3) modulating the postoperative tumor microenvironment. To this end, we have designed a reactive oxygen species (ROS)-sensitive biphasic drug-release system that integrates the 3 aspects. This PIDDS is composed of free TMZ, free Cur, and drug-loaded (Cur and TMZ) PLGA nanoparticles (NPs) incorporated into an ROS-sensitive and thermo-sensitive gel (Scheme 1). Firstly, we established a postoperative recurrence model of glioma. We prepared PLGA NPs co-loaded with Cur and TMZ and incorporated free TMZ, free Cur, and the drug-loaded nanoparticles into the ROS-sensitive chitosan/gelatin thermo-sensitive gel to construct the PIDDS. After surgical resection of the brain tumor, the drug-loaded thermo-sensitive gel is immediately injected into the resection cavity in situ. The gel then undergoes thermo-gelling to form a structure that fits the resection cavity. The free TMZ and free Cur in gel can rapidly release, completing the first phase of rapid drug release. This initial release works synergistically to kill a large number of residual tumor cells and simultaneously reduces the postoperative tumor response, thereby initially improving the postoperative tumor microenvironment. As the ROS-sensitive gel gradually degrades, the PLGA NPs would be released from the gel and slowly release Cur and TMZ, completing the second phase of sustained drug release. Cur suppresses the stemness of residual GSCs and continuously improves the postoperative tumor microenvironment. It also works synergistically with TMZ to inhibit the proliferation of residual tumor cells. Ultimately, this biphasic drug-release system achieves long-term inhibition of GBM recurrence, providing a new therapeutic strategy and approach for the precise postoperative treatment of GBM.

## 2 Materials and methods

### 2.1 Materials

Rat glioma C6 cells and Luciferase labeled C6 cells (Luc—C6) were purchased from Opcell Biotechnology, China. TMZ, Cur, PLGA_75/25_, PNIPAAm-grafted chitosan (20 % grafting degree), gelatin, chitosan, and cysteine were purchased from Meilun Biotechnology, China. The Seahorse XF e96 analyzer, mitochondrial stress test kit, and glycolysis stress test kit were purchased from Agilent Technologies, USA. All other reagents were of analytical grade. Male SD rats were purchased from Wushi Animal and fed in SPF condition. All animal procedures were conducted in accordance with the requirements of the Animal Ethics Committee of Fuzhou University Affiliated Provincial Hospital.

### 2.2 Network pharmacology

#### 2.2.1 Drug targets screening

Using “Cur” and “TMZ” as keywords, the standard structures and Simplified Molecular Input Line Entry System (SMILES) notations of Cur and TMZ were queried in the PubChem database (https://pubchem.ncbi.nlm.nih.gov/). The SMILES notations of the drugs were then used to predict their targets through the Swiss Target Prediction database (http://www.swisstargetprediction.ch/). The prediction results were filtered based on a Probability value greater than 0.6. A total of 100 unique targets were obtained for TMZ, and 100 unique targets were obtained for Cur.

#### 2.2.2 GSCs and glioma cell targets screening

Using “glioma stem cells” as the keyword, human gene was screened in GeneCards database (with Relevance score greater than 30, https://www.genecards.org/), OMIM database (https://www.omim.org/), and Open Targets database (https://platform.opentargets.org/). After merging and removing duplicates from these 3 databases, a total of 3633 disease-related genes were identified. The top 1500 genes were selected based on their Relevance score.

Using “glioma” as the keyword, human gene was screened in GeneCards database (with Relevance score greater than 30, https://www.genecards.org/), OMIM database (https://www.omim.org/), and Open Targets database (https://platform.opentargets.org/). After merging and removing duplicates from these 3 databases, a total of 10,019 disease-related genes were identified. The top 1500 genes were selected based on their Relevance score.

#### 2.2.3 Venn diagram

The selected drug targets and disease targets were input into the Venny 2.1 software for creating a Venn diagram, and suitable targets were identified as predicted targets for the drugs to act on the disease for subsequent pathway enrichment analysis.

#### 2.2.4 Protein-protein interaction (PPI) network construction

The common targets of the drug and disease were input into the STRING database (https://string-db.org/cgi/input.pl) to construct a PPI network, with the organism set as “homo sapiens”.

#### 2.2.5 Gene Ontology (GO) enrichment analysis

The common targets of the drug and disease, as well as the disease targets, were subjected to GO enrichment analysis in terms of biological process (BP), molecular function (MF), and cellular component (CC). The STRING database was referenced for this analysis. Items with an adjusted *P*-value ≤0.05 were selected. R 4.4.0 was used, with the packages clusterProfiler, enrichplot, and ggplot2 installed and imported, to create bubble plots.

#### 2.2.6 *Kyoto Encyclopedia of Genes and Genomes (KEGG)* enrichment analysis

The common targets of the drug and disease, as well as the disease targets, were subjected to KEGG pathway enrichment analysis. The STRING database was referenced for this analysis. Items with an adjusted P-value ≤0.05 were selected. R 4.4.0 was used, after installing and loading the clusterProfiler package, bar plots were created.

### 2.3 PLGA NPs preparation

TMZ and Cur were loaded into nanoparticles with the following formulation composition by the solvent evaporation method: 30 mg of PLGA_75/25_, 20 mg of TMZ, and 5 mg of Cur were dissolved in 1 mL dichloromethane. Then, 2 mL of a 5 % PVA solution was added and vigorously agitated. The mixture was subjected to probe sonication (200 W, intermittent sonication, 1 s-on and 1 s-off) for 30 cycles under ice bath condition to prepare the primary W/O emulsion. The resulting emulsion was slowly dispersed dropwise into 8 mL of a 5 % PVA emulsifier solution. The mixture was stirred magnetically at room temperature for 1 h (250 rpm) and then rotary evaporated under reduced pressure at 40 °C for 20 min to remove the organic solvent. After centrifugation at 4 °C (18,000 ×g, 30 min), the supernatant was discarded, and the nanoparticles were re-dispersed in PBS. The centrifugation and washing steps were repeated twice to remove free drugs that were not loaded into the nanoparticles. Entrapment efficiency (EE) and drug loading (DL) capacity of TMZ/Cur in PLGA NPs were tested by HPLC.

### 2.4 Gel PIDDS preparation

Disulfide bonds were selected as the ROS-sensitive groups, and poly(N-isopropylacrylamide) (PNIPAAm) was chosen as the thermo-sensitive polymer to enable the drug-loaded gel to undergo temperature-sensitive gelling in the GBM resection cavity, releasing PLGA NPs and free drugs in the ROS-enrichment area. One gram of chitosan and 0.25 g of gelatin were weighed and dissolved in 40 mL of ultrapure water containing 0.1 M acetic acid by continuous stirring at 50 °C for 2 h. Insoluble materials were remo*v*ed by centrifugation at 5000 rpm for 15 min at 4 °C to prepare the chitosan/gelatin solution, which was then sterilized by autoclaving. Sterilized PNIPAAm-grafted chitosan solution (20 % grafting degree) was added and mixed thoroughly. Cysteine, free drugs, and drug-loaded PLGA NPs were added, and the mixture was stirred until completely dissolved. In an ice bath environment, a sterile mild crosslinking agent (H₂O₂, with a final concentration of 0.01 % *v*/v) was added to the mixture, which was then continuously stirred in the ice bath for 2 h to form a crosslinked network. The mixture was then transferred to a 37 °C environment, where the PNIPAAm underwent hydrophobic aggregation and phase transition, and the oxidative crosslinking reaction of disulfide bonds was accelerated at body temperature. After resting for 5 min, the temperature-sensitive phase transition and disulfide bond crosslinking worked together to form a stable dual-sensitive (temperature + ROS) hydrogel network that encapsulated the free drugs and PLGA NPs. The gel was washed with sterile deionized water to remove unreacted crosslinking agents and impurities. The washed gel could be stored in PBS at 4 °C for later use. All steps (solution preparation, addition of components, crosslinking, and washing) were performed under sterile conditions.

### 2.5 In vitro characteristics of PIDDS

The particle size and zeta potential of PLGA NPs were evaluated, and transmission electron microscopy (TEM) observation of NPs was conducted. Scanning electron microscopy (SEM) image of the gel were taken at 3 scales: 500 μm, 50 μm, and 5 μm, to observe the gel structure and the attachment of NPs. Free drugs were used as controls, drug-loaded gels (gels without ROS groups referred to as Gels, and gels with ROS groups referred to as ROS-Gels) were added to 50 mL of ROS release medium (50 mL pH 7.4 PBS + 100 μM H₂O₂ + 0.5 % SDS) and placed in brown vials. These were incubated at 37 °C with shaking at 100 rpm. At various time points, the entire release medium was removed and replaced with fresh ROS medium solution. The in vitro drug release behavior of the gel-based PIDDS in the ROS medium environment was measured, and cumulative drug release curves were plotted.

The changes in important groups of gels (Gels and ROS-Gels) were detected by Fourier transform infrared spectroscopy (FTIR) with the wavenumber range set at 500–4000 cm^−1^. The phase transition points of gels were detected by differential scanning calorimetry (DSC) with the temperature range set at 4–50 °C.

### 2.6 GSCs inhibition and CCK-8 assay

GSC spheroids: C6 cells were screened and cultured long-term in stem cell medium, which consisted of DMEM/F12 (1:1), epidermal growth factor (20 μg/L), basic fibroblast growth factor (20 μg/L) and B27 (1:50). Immunofluorescence staining was used to detect the GSCs marker CD133 labeled with Cy5.5 to confirm the formation of GSC spheroids.

TMZ/Cur inhibition of GSCs cell proliferation: The CCK-8 assay was used with a detection wavelength of 450 nm. GSC spheroids were digested with trypsin, re-counted, and plated in a 96-well plate using DMEM medium. On the second day, cells were treated with drugs to determine the toxicity of TMZ and Cur individually on GSCs and to calculate their respective IC_50_ values. Subsequently, TMZ was combined with Cur at its IC_25_ concentration (the drug concentration that inhibiting 25 % of tumor cell proliferation, 4 μM) to assess the combined toxicity of the 2 drugs on GSCs. Furthermore, TMZ and Cur at the IC_25_ concentration were loaded into PLGA NPs to analyze the effects of the nano-system on drug-mediated tumor cell killing through CCK-8 assay.

TMZ/Cur Inhibition of GSCs spheroids: After co-incubating different drug groups (TMZ and Cur) with CD133+ GSC spheroids, immunofluorescence staining was used to detect the expression of CD133 labeled with Cy5.5.

### 2.7 Measurement of cellular glycolysis and mitochondrial respiration levels

Cells were seeded into the Seahorse XFe96 cell culture plate, with an appropriate volume of cell suspension added to each well to achieve a cell density of approximately 2 × 10^4^ cells per well. The cell culture medium was replaced with Seahorse XF medium to eliminate the effects of serum and sodium bicarbonate. The cells were then co-incubated with either TMZ alone (195.9 μM), Cur (4 μM), or a combination of 195.9 μM TMZ and 4 μM Cur. The cell culture plate was placed into the Seahorse XF instrument, and the measurements were conducted according to the preset program. The instrument automatically injected the pre-set reagents and monitored the changes in extracellular acidification rate (ECAR) and oxygen consumption rate (OCR) in real time.

### 2.8 ECAR measurement

Basal glycolysis: after the addition of 10 mM glucose, cells begin glycolysis, producing protons that lead to an increase in ECAR. The ECAR value at this point reflects the cell's basal glycolytic capacity. Maximal glycolytic capacity: upon the addition of 1 μM oligomycin, mitochondrial ATP production is inhibited, forcing the cells to generate energy through glycolysis. The ECAR increases further, and the ECAR value at this point reflects the cell's maximal glycolytic capacity. Glycolytic reserve capacity: the difference between maximal glycolytic capacity and basal glycolysis represents the glycolytic reserve capacity. Non-glycolytic acidification: finally, the addition of 50 mM 2-deoxyglucose (2-DG) inhibits glycolysis. The ECAR value at this point reflects the level of non-glycolytic acidification.

### 2.9 OCR measurement

Basal Respiration: without adding any drugs, cells perform oxidative phosphorylation through the mitochondrial respiratory chain, consuming oxygen to produce ATP. The OCR value at this point reflects the cell's basal respiration capacity, which is the oxygen consumption level of mitochondria under normal physiological conditions. ATP production: upon the addition of 1 μM oligomycin, the activity of ATP synthase is inhibited, preventing proton backflow into the mitochondrial matrix and thus inhibiting mitochondrial ATP synthesis. Since ATP synthesis is blocked, the proton motive force across the mitochondrial respiratory chain increases, leading to reduced mitochondrial respiration and a decrease in OCR. The decrease in OCR at this point reflects the portion of mitochondrial respiration used for ATP synthesis, i.e., the ATP production capacity. Maximal respiration: after adding FCCP, the proton motive force across the mitochondrial membrane is dissipated, removing the constraints on the mitochondrial respiratory chain, and the mitochondria reach their maximal respiration capacity, with OCR reaching peak. The OCR value at this point reflects the maximal respiration capacity of mitochondria when not limited by ATP synthase, i.e., the maximal oxidative phosphorylation capacity of mitochondria. Mitochondrial reserve Capacity: The difference between maximal respiration and basal respiration is the mitochondrial reserve capacity. It reflects the additional respiratory capacity that cells can utilize under stress conditions, i.e., the potential respiratory capacity of mitochondria between normal physiological conditions and maximal respiration. Non-mitochondrial respiration: finally, by adding rotenone and antimycin A, the electron transfer in the mitochondrial respiratory chain is inhibited, completely blocking mitochondrial oxygen consumption. The remaining OCR value at this point reflects oxygen consumption from non-mitochondrial sources, such as the activity of oxidases in the cytoplasm or redox enzymes on the cell membrane, i.e., the level of non-mitochondrial respiration.

By comparing the differences in ECAR and OCR values between different drug-treated groups and the control group, the effects of the drugs on the glycolytic level and mitochondrial respiration of tumor cells can be analyzed.

### 2.10 Western blot

C6 cells and glioma cells derived from digested GSCs spheroids were cultured separately. When the cell confluence reached approximately 50 %, they were co-incubated with TMZ alone (195.9 μM), Cur alone (4 μM), and a combination of 195.9 μM TMZ and 4 μM Cur. After 24 h of drug treatment, protein extraction was performed. Protein quantification was carried out using the BCA method, and Western blot analysis was conducted with β-actin as the internal reference. The primary antibodies selected included: EFGR, CHEK1, JAK1, JAK2, CD133, TLR9, MMP13, CA9, and GSK3B.

### 2.11 Pharmacodynamics

Construction of rat intracranial glioma model: luciferase-labeled GSCs cells (Luc-GSCs) with a density of 70–80 % were subjected to trypsin digestion, washed twice with PBS, and resuspended to a cell density of 1 × 10^8^ cells/mL. Male SD rats were fasted and deprived of water for 12 h before tumor cells implantation. After anesthesia with isoflurane, the hair on the top of the head was shaved to expose the scalp, and the rats were fixed on a stereotactic apparatus. After disinfection with 75 % alcohol, a midline incision was made on the top of the head parallel to the rat's body to expose the bregma. Taking the midpoint of the bregma as the origin, a 5 mm diameter circular opening was drilled through the skull 2 mm to the right of the sagittal suture and 2 mm to the back from the coronal suture to expose the dura mater. A 5 μL micro syringe fixed on the stereotactic apparatus was used to vertically penetrate the dura mater to a depth of 3.5 mm. After maintaining for 1 min, the needle was retracted by 1 mm and maintained for another 1 min, followed by the slow injection of 5 μL of Luc-GSCs cell suspension in serum-free PBS (5 × 10^5^ cells). The needle was left in place for 5 min, then retracted by 1 mm and maintained for 1 min before slowly withdrawing the needle. The scalp was sutured, and 5 % ampicillin sodium was administered intraperitoneally at a dose of 250 mg/kg to prevent surgical infection.

Glioma Resection Surgery: on the 7th day of tumor growth, in vivo imaging was performed to confirm the tumor growth and the animal's condition for tumor resection surgery. The SD rats with the constructed glioma model were fasted and deprived of water for 12 h before surgery. After anesthesia with isoflurane, the hair on the top of the head was removed, and the scalp was re-opened to expose the original 5 mm circular incision, revealing the growing glioma. The tumor resection surgery was performed, avoiding blood vessels to prevent major bleeding, until normal brain tissue was visible to the naked eye. After the surgery, the scalp was sutured, and ampicillin sodium was administered to prevent surgical infection.

Animal grouping and dosing: healthy male SD rats were randomly divided into 5 groups, with 9 rats in each group: Resection only group (PBS injection after tumor resection), Free TMZ group (free TMZ injection after tumor resection), Free TMZ/Cur group (free TMZ and Cur injection after tumor resection), Gels group (drug-loaded non-ROS-sensitive gel PIDDS after tumor resection), and ROS-Gels group (drug-loaded ROS-sensitive gel PIDDS after tumor resection). The dosing of TMZ was 20 mg/kg, and the dosing of Cur was 5 mg/kg. The ratio of free drugs to drugs loaded into PLGA NPs was 1:1.

Pharmacodynamics: after confirming that the animals had recovered from anesthesia following the brain tumor resection surgery and drug administration, living imaging was performed on day 8 to confirm the tumor resection ratio and residual size. Subsequent in vivo imaging was conducted every 7 days to observe tumor recurrence until the animals died naturally. Survival rate and animal weight were used as indicators to determine the effective dose and investigate the dose-effect relationship, evaluating the therapeutic effect of the combination of the 2 drugs in inhibiting tumor recurrence.

### 2.12 Hematoxylin and eosin (H&E) staining and TUNEL staining

On day 14 after GSCs inoculation, 3 rats from each group were euthanized by cardiac perfusion. The heart, liver, spleen, lung, kidney, and left cerebral hemisphere were harvested to prepare tissue sections and perform H&E staining to determine the toxicity of the drugs on various tissues. Tissue sections were also prepared from the recurrent tumors on day 14 and stained with TUNEL (using a DAB detection system, with apoptotic cells appearing brown) to verify the cytotoxic effects of the drugs on tumor cells, particularly their ability to induce apoptosis.

### 2.13 Statistics

All tests were performed in triplicate, and the results were expressed as the mean ± standard deviation. The differences between 2 group means were determined using the unpaired Student's *t*-test. For multiple comparisons, two-way analysis of variance (ANOVA) was used to test for statistical significance. All statistical calculations were completed using GraphPad Prism 9.0 software.

## 3 Results and discussion

### 3.1 In vitro characteristics of PIDDS

**Fig. 1:**
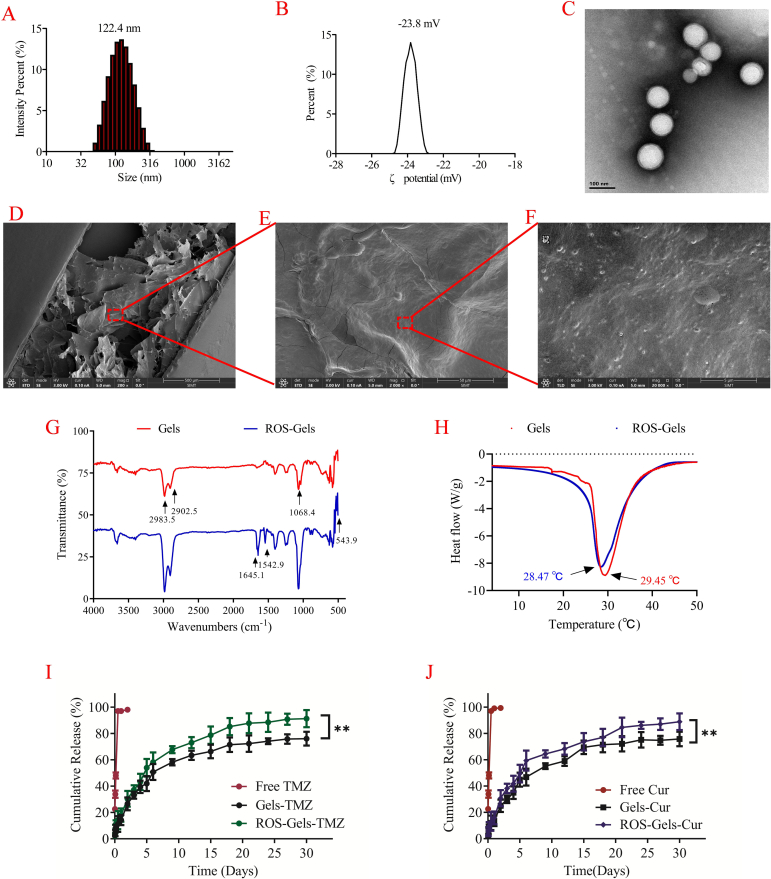
In vitro characterization of the Gel PIDDS. A: Average particle size of PLGA NPs. B: Surface charge of PLGA NPs. C: TEM characterization of PLGA NPs. D: SEM of ROS-Gels, scale bar: 500 μm. E: SEM of ROS-Gels, scale bar: 50 μm. F: SEM of ROS-Gels, scale bar: 5 μm. G: FTIR spectrum of Gels and ROS-Gels. H: DSC of Gels and ROS-Gels. I: Release of TMZ in ROS release medium. J: Release of Cur in ROS release medium.

The TMZ/Cur co-loaded PLGA NPs prepared by the emulsion solvent evaporation method exhibited favorable physical properties (Fig. 1A-C). Dynamic light scattering (DLS) analysis showed that the average hydrodynamic particle size of the drug-loaded NPs was 122.4 ± 10.5 nm (PDI < 0.2) (Fig. 1A), indicating uniform nanoparticle size and an ideal range for tumor tissue penetration (< 200 nm). The surface ζ-potential measurement was −23.8 ± 2.6 mV (Fig. 1B), with a higher negative charge contributing to the colloidal stability of the NPs and reducing non-specific protein adsorption. Accordingly, the EE and DL ability of PLGA NPs were 79.8 % ± 1.8 % and 4.9 % ± 0.4 % for Cur, 66.5 % ± 3.3 % and 10.7 % ± 1.1 % for TMZ, respectively (Supplemental Table 1). TEM images (Fig. 1C) further confirmed that the NPs were regular spherical shapes with a particle size distribution consistent with the DLS results and no significant aggregation. These results indicated the successful construction of a highly stable nanoparticle delivery system suitable for subsequent gel drug loading.

SEM was used to observe the morphology of the drug-loaded gel at 3 scales (500 μm, 50 μm, 5 μm) (Figs. 1D-F). At low magnification (500 μm), the gel exhibited a layered porous network structure (Fig. 1D), which is conducive to drug release. At medium magnification (50 μm), the gel surface showed abundant folds and uneven structures (Fig. 1E), increasing the specific surface area. The critical high-magnification image (5 μm) (Fig. 1F) clearly showed a large number of PLGA NPs uniformly attached to the surface and inside the pores of the gel fibers. This multi-scale porous framework mimics the extracellular matrix, providing an ideal biocompatible filling carrier for the postoperative resection cavity.

FTIR was used to further verify the successful construction of the drug-loaded gels and the introduction of ROS-sensitive groups (Fig. 1G). The results showed that both Gels and ROS-Gels retained the characteristic peaks of the basic backbone of chitosan/gelatin gel matrix (such as C-O-C stretching vibration at approximately 1068.4 cm^−1^, C—H stretching vibration at approximately 2902.5 cm^−1^ and 2983.5 cm^−1^), as well as the characteristic peaks of the drug and PLGA NPs, indicating that the drug-loading process did not destroy the chemical structure of the main body of the gel. The key finding was that in the infrared spectrum of ROS-Gels, the characteristic absorption peaks of PNIPAAm were clearly observed, including the amide I band (C=O stretching vibration, 1645.1 cm^−1^) and the amide II band (N—H bending vibration, approximately 1542.9 cm^−1^), confirming the successful grafting of the thermosensitive polymer PNIPAAm. More importantly, a characteristic peak for the stretching vibration of disulfide bonds (-S-S-) appeared at 543.9 cm^−1^, which was only present in ROS-Gels, conclusively proving that the ROS-sensitive crosslinker cysteine was successfully incorporated into the gel network structure through disulfide bonds.

DSC was used to evaluate the temperature sensitivity of the gels (Fig. 5H). Both Gels and ROS-Gels exhibited typical thermosensitive phase transition behavior. The phase transition temperature of Gels was 29.45 °C (corresponding to a heat flow valley of −8.7 W/g), while that of ROS-Gels was 28.47 °C (corresponding to a heat flow valley of −8.2 W/g). The phase transition temperatures of both were slightly lower than the human physiological temperature (37 °C), which is of vital importance: it ensures that the gels, prepared and stored at room temperature, are in an injectable sol state, and after being injected into the postoperative resection cavity of the brain (37 °C), they can quickly undergo a phase transition to a stable gel state, thus fitting the shape of the resection cavity and achieving in situ retention.

In the ROS release medium (containing 100 μM H₂O₂) that simulated the postoperative tumor microenvironment, the drug release kinetics of non-ROS-sensitive gels (Gels) and ROS-sensitive gels (ROS-Gels) were systematically compared (Fig. 1I**&J**). In the Gels group, both TMZ and Cur exhibited rapid release in the first 5 days and slow sustained release in the following 25 days, with cumulative release rates of 76.1 ± 5.3 % (TMZ) and 75.8 ± 5.5 % (Cur) after 30 days, indicating compatible release behavior of the 2 drugs in the gel. In the ROS-Gels group, the drug release was significantly increased (*p* < 0.01), with cumulative release rates of 91.3 ± 6.5 % (TMZ) and 88.8 ± 6.3 % (Cur) after 30 days, indicating that the ROS-sensitive disulfide bond crosslinking effectively responded to H₂O₂ stimulation, promoting gel degradation and drug diffusion. Notably, both gels exhibited an initial burst release of about 40–50 % within the first 120 h, attributed to the rapid diffusion of free drugs in the gel, consistent with the designed “biphasic drug release” strategy: free drugs rapidly killed residual tumor cells after surgery, while NPs were controlled released by the ROS-sensitive gel to achieve long-term inhibition of GBM recurrence.

This study successfully constructed an ROS-responsive biphasic drug-release PIDDS, and the in vitro characterization fully validated the rationality and feasibility of the design. The particle size and surface charge of the PLGA NPs endowed them with good tumor penetration and stability, laying the foundation for efficient drug delivery to GSCs. FTIR analysis confirmed the successful incorporation of ROS-sensitive groups (disulfide bonds) and thermosensitive polymers (PNIPAAm) into the gel network, providing a molecular basis for the dual functions of “ROS responsiveness” and “temperature sensitivity.” The DSC results further showed that the phase transition temperature of the gels (∼29 °C) was slightly lower than body temperature, ensuring rapid sol-gel transition upon injection into the resection cavity and achieving in situ fixation. The three-level porous structure revealed by SEM images (Figs. 1D-F) has 3 advantages: 1) Mechanical compatibility: the layered network provides mechanical support and fits the shape of the surgical cavity; 2) Drug-loading inclusiveness: the porous framework efficiently loads free drugs and NPs; 3) Drug-release controllability: the pore channels, in conjunction with ROS-sensitive groups, regulate the drug release rate.

Clinical studies (Grzmil et al., 2025) and our previous research (Chen et al., 2022) have confirmed that the postoperative resection cavity of GBM has a hypoxic-ROS-enriched area, where ROS-sensitive gel can selectively degrade and release free drugs and PLGA NPs, increasing the local concentration of drugs at the recurrent site while reducing systemic exposure toxicity. The release profiles confirmed the ROS-responsive behavior of the gel system, with significantly higher cumulative release from ROS-Gels compared to ordinary gels in H₂O₂-containing medium (TMZ: 91.3 % vs 76.1 %; Cur: 88.8 % vs 75.8 %), demonstrating successful disulfide bond-based degradation (Figs. 1I**&J**). During the critical initial 120-h (5-day) rapid release phase—a key window for targeting residual cells—approximately 45 % of TMZ and 50 % of Cur were released from the ROS-gel. Based on the total drug loading, this corresponded to a cumulative release of 1.8 mg of TMZ (489.1 μM) and 0.5 mg of Cur (27.2 μM). These released concentrations far exceed the IC₅₀ of the free drug combination (195.9 μM TMZ + 4 μM Cur), indicating that the burst release creates a highly cytotoxic zone around the resection cavity capable of eliminating most residual tumor cells.

This biphasic release strategy enables rapid clearance of residual cells through immediate free drug release, followed by sustained nanoparticle-mediated targeting of GSCs and the tumor microenvironment (e.g., via CA9/TLR9 inhibition), thereby achieving long-term control. This extended-release profile addresses the major limitation of short-term release systems such as Gliadel® wafers (3–4 days). Overall, the in vitro characterization confirms that the PIDDS possesses optimal physicochemical properties, structural integrity, and microenvironment-responsive release capability, providing a solid foundation for its anti-tumor efficacy in subsequent in vivo experiments.

### 3.2 Network pharmacology

#### 3.2.1 enn and PPI network

**Fig. 2:**
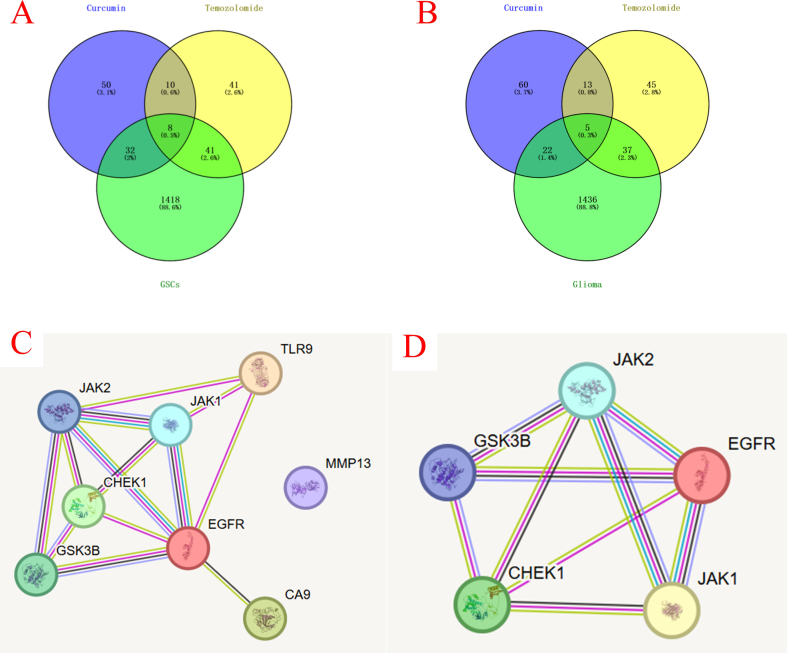
Venn diagrams and PPI networks of the targets of TMZ/Cur on GSCs and glioma cells using network pharmacology. A: Venn diagram of the TMZ/Cur targeting on GSCs. B: Venn diagram of the targets of TMZ/Cur targeting on glioma. C: PPI network of TMZ/Cur on GSCs. D: PPI network of TMZ/Cur on glioma.

The Venn diagram results are shown in Figs. 2A & B. Fig. 2A depicted the 8 common targets (TLR9, EGFR, GSK3B, CA9, CHEK1, MMP13, JAK1, JAK2), identified for the combination of Cur and TMZ against GSCs. This indicated that the combination therapy has broad target coverage when targeting GSCs, potentially affecting multiple key biological processes of GSCs. Fig. 2B showed the 5 common targets (EGFR, GSK3B, CHEK1, JAK1, JAK2) identified for the combination of Cur and TMZ against glioma. These targets partially overlap with those of GSCs but are fewer in number, suggesting that in the overall glioma cell population, the targets of the combination therapy are relatively concentrated. The overlapping targets (EGFR, GSK3B, CHEK1, JAK1, JAK2) may be the common key targets in both GSCs and the overall glioma cell population, and they may play important roles in the occurrence, development, and drug resistance formation of glioma.

The PPI network results were shown in Figs. 2C & D. Fig. 2C illustrated the PPI network of the TMZ/Cur combination against GSCs, comprising 8 nodes and 13 edges. The complex interactions among these targets formed a relatively tight PPI network. This complex network may imply that the combination therapy can intervene in the biological behavior of GSCs from multiple angles, thereby enhancing the therapeutic effect. Fig. 2D showed the PPI network of the TMZ/Cur combination against glioma, containing 5 nodes and 9 edges. Although the number of nodes was smaller, the number of edges was relatively high, indicating tight interactions among these targets. This may suggest that in the overall glioma cell population, the mechanism of action of the combination therapy is relatively focused, mainly revolving around a few key targets. This relatively concentrated network structure may imply that the combination therapy acts more directly on the glioma cell population, rapidly affecting key signaling pathways to exert its therapeutic effect.

#### 3.2.2 GO enrichment analysis

**Fig. 3:**
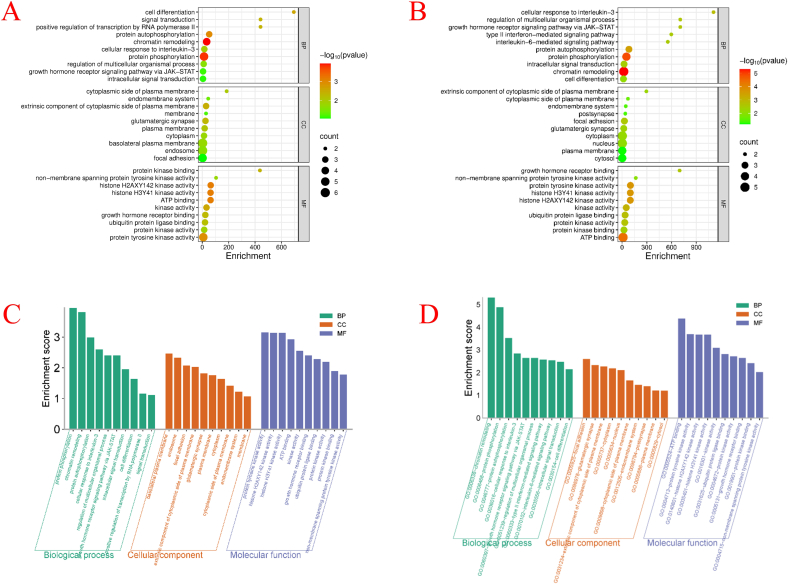
GO and KEGG enrichment analysis of TMZ/Cur combination on GSCs and glioma cells. A: GO enrichment analysis of TMZ/Cur combination on GSCs; B: GO enrichment analysis of TMZ/Cur combination on glioma cells; C: KEGG enrichment analysis of TMZ/Cur combination on GSCs; D: KEGG enrichment analysis of TMZ/Cur combination on glioma cells.

To further explore the potential mechanisms of TMZ/Cur combination in treating GSCs, we performed GO functional enrichment analysis on the intersecting targets identified, covering 3 dimensions: biological process (BP), cellular component (CC), and molecular function (MF). The results are shown in Figs. 3A & B. For GSCs (Fig. 3A), in terms of BP, the target genes were significantly enriched in 3 pathways: cell differentiation, signal transduction, and positive regulation of transcription by RNA polymerase II. In terms of CC, the target genes were significantly enriched in the cytoplasmic side of the plasma membrane pathway. In terms of MF, the target genes were significantly enriched in protein kinase binding. This revealed that the potential synergistic mechanism of TMZ/Cur in inhibiting GSCs mainly revolved around 3 core aspects: inducing differentiation, interfering with the signal transduction hub on the inner side of the plasma membrane (especially by binding to and inhibiting key protein kinases), and regulating gene transcription (Diki et al., 2025). Combining the results of the Venn diagram and PPI analysis, this potential synergistic mechanism can be divided into 3 levels: spatial level, targeting the cytoplasmic side of the plasma membrane (EGFR/JAK/TLR9) signaling hub to block oncogenic signaling (Qu et al., 2024); functional level, inhibiting kinases (EGFR/JAK/CHEK1) to block GSC cell survival/repair pathways, reprogramming transcription (JAK-STAT/TLR9-NF-κB) to downregulate stemness genes, and inducing differentiation (GSK3B) to deplete stem cells (Gersey et al., 2017); microenvironment level, disrupting the adaptability and invasiveness of GSCs by inhibiting CA9/MMP13 (Sarkar et al., 2024).

For glioma cells (Fig. 3B), in terms of BP, the target genes were significantly enriched in 5 pathways: cellular response to interleukin-3, regulation of multicellular organismal process, growth hormone receptor signaling pathway via JAK-STAT, type ll interferon-mediated signaling pathway, and interleukin-6-mediated signaling pathway. In terms of CC, the target genes were significantly enriched in the extrinsic component of the cytoplasmic side of the plasma membrane pathway. In terms of MF, the target genes were significantly enriched in growth hormone receptor binding. This revealed that the core molecular mechanism of TMZ/Cur in inhibiting glioma cells lied in the synergistic regulation of tumor-related tumor/immune signaling (especially IL-6, IFN-γ, IL-3) and growth factor signaling. Combining the results of the Venn diagram and PPI analysis, the core of the synergistic anti-GSCs/glioma effect of TMZ/Cur was to disrupt the stemness maintenance signaling of GSCs, the DNA repair barrier of glioma cells, and growth factor pathways through 5 key targets (EGFR/GSK3B/CHEK1/JAK1/JAK2): specifically, inhibiting the JAK1/2-STAT3 pathway to dismantle the stemness of GSCs, blocking EGFR signaling to suppress tumor proliferation/invasion, activating GSK3B to degrade β-catenin and promote apoptosis, and inhibiting CHEK1 to cause DNA repair failure, thereby enhancing TMZ toxicity. This multi-target mechanism explained the biological basis of the JAK-STAT pathway, growth factor signaling, and tumor response enriched in the previous GO analysis and provided a theoretical basis for clinically reversing glioma (especially stem cells) drug resistance.

#### 3.2.3 KEGG enrichment analysis

To further elucidate the potential molecular mechanisms of the TMZ/Cur combination in inhibiting GSCs/glioma, we performed KEGG pathway enrichment analysis on the intersecting targets identified. The results (Figs. 3C & D) showed that, for both GSCs and glioma cells, 6 pathways including protein phosphorylation, chromatin in the nucleus, protein kinase activity, H2AXY142 kinase activity, H3Y41 kinase activity, and ATP binding, were significantly enriched. Combining the 8 common targets identified for the TMZ/Cur combination against GSCs (TLR9, EGFR, GSK3B, CA9, CHEK1, MMP13, JAK1, JAK2) and the 5 common targets identified against glioma cells (EGFR, GSK3B, CHEK1, JAK1, JAK2), the molecular mechanisms of TMZ/Cur in inhibiting GSCs and glioma cells can be divided into the following 3 core aspects:

1) Core common mechanism - synergistic targeting of DNA repair, kinase signaling, and epigenetics by the drug combination: this involved disrupting DNA damage repair (CHEK1 + H2AXY142 regulation) (Fu et al., 2025), inhibiting the JAK/STAT-epigenetic hub (JAK1/JAK2 + H3Y41 regulation), and blocking the EGFR-GSK3β-Wnt proliferation axis (Schlange et al., 2007).
2) GSCs-specific mechanism - synergistic targeting of hypoxia adaptation, immune evasion, and invasion by the drug combination: this included reversing the hypoxic microenvironment (CA9) (Geiß et al., 2021), relieving immune suppression (TLR9) (Merrell et al., 2006), and inhibiting invasion and metastasis (MMP13) (Li et al., 2022).
3) Energy metabolism and global regulation - synergistic ATP-competitive inhibition and repair exhaustion by the drug combination: this activated a global blockade of the stress response capabilities of GSCs/glioma cells.

In summary, the results of the GO and KEGG analyses revealed that the TMZ/Cur combination may exert a “three-in-one” multi-pathway and multi-mechanism synergistic anti-GSCs/glioma effect through a triaxial collaborative drive, namely: 1) regulating the tumor proliferation-stemness exhaustion axis; 2) regulating the DNA repair collapse axis, and 3) regulating the microenvironment reprogramming axis. These aspects supported the potential application of this combination strategy in glioma treatment, enabling Cur to reshape the tumor microenvironment in depth by secreting inhibitory factors, thereby blocking signal transduction and then silencing epigenetic genes. This enhanced the genotoxicity of TMZ from “unidirectional killing” to a multi-dimensional collaborative clearance mode involving “immunity-epigenetics-metabolism.” While Sections 3.2.2 and 3.2.3 focused on elucidating the intrinsic synergistic mechanisms and shared molecular targets between TMZ and Cur through network pharmacology, it is important to emphasize that the PLGA NPs and PIDDS were not included in this bioinformatic analysis. This is because NPs and PIDDS serve primarily as a pharmacologically inert delivery vehicles designed to enhance drug stability, control release kinetics, and improve cellular uptake rather than directly engaging with specific biological pathways.

### 3.3 GSC inhibition and CCK-8 assay

**Fig. 4:**
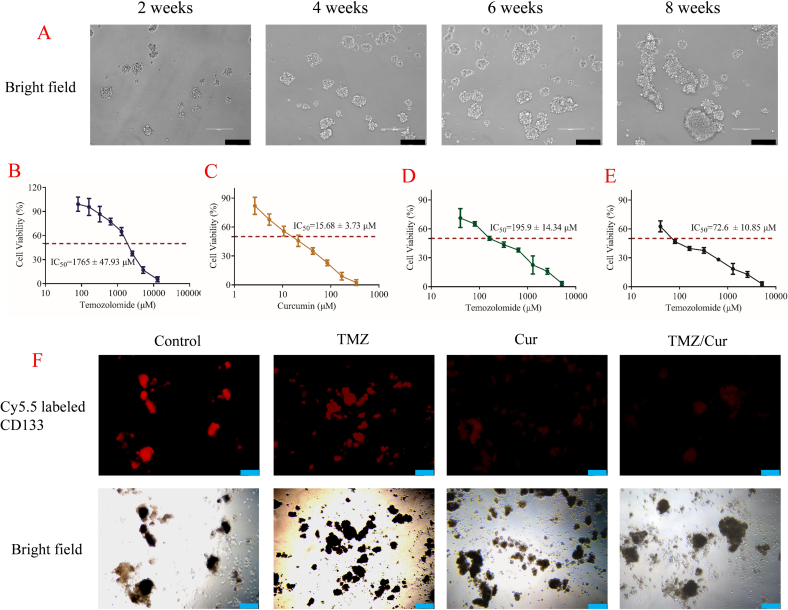
Construction of GSCs spheroids and the inhibitory effects of TMZ/Cur Combination on GSCs spheroids. A: Successful construction of GSCs spheroids within 8 weeks; B: CCK-8 results of TMZ alone; C: CCK-8 results of Cur alone; D: CCK-8 results of TMZ/Cur combination; E: CCK-8 results of TMZ/Cur co-loaded into PLGA NPs; F: TMZ/Cur combination inhibiting GSCs spheroids, resulting in decreased expression of the stem cell marker CD133 labeled with Cy5.5.

To evaluate the effects of TMZ and Cur alone and their combination on the anti-proliferative activity of GSCs, we established a rat C6 glioma-derived GSCs spheroid model. As shown in Fig. 4A, C6 cells cultured in stem cell medium containing growth factors began to form tumor spheroids from the second week, which gradually increased in size and fused over time, forming typical large GSCs tumor spheroids by the 8th week. Immunofluorescence staining confirmed that the formed tumor spheroids highly expressed the GSC marker CD133 (labeled with Cy5.5) (Fig. 4F), verifying the successful establishment of the GSCs model.

Subsequently, we assessed the cytotoxic effects of TMZ and Cur on GSCs using CCK-8 assay. The results showed that TMZ alone had limited inhibitory effects on GSCs, with an IC_50_ value as high as 1765 ± 47.93 μM (Fig. 4B) (Zhao et al., 2021), significantly higher than the reported IC_50_ values for differentiated glioma cells. This clearly confirmed the strong intrinsic resistance of GSCs to the first-line chemotherapeutic drug TMZ. In sharp contrast, Cur alone exhibited significant inhibitory activity against GSCs proliferation (Fig. 4C), with an IC_50_ value of 15.68 ± 3.73 μM, indicating that Cur itself has the potential to target the stemness of GSCs and inhibit their growth.

Given that our previous network pharmacology analysis suggested synergistic targets for TMZ and Cur (such as CHEK1, JAK/STAT, and EGFR), we selected the IC_25_ concentration of Cur (4 μM) as the combination dose to further evaluate the combined effects of the 2 drugs. As shown in Fig. 4D, when 4 μM Cur was combined with TMZ, the cytotoxicity of TMZ against GSCs was significantly enhanced, with its IC_50_ value dramatically reduced to 195.9 ± 14.34 μM, nearly a 9-fold decrease compared to TMZ alone (*p* < 0.001). This result strongly confirmed that low-dose Cur can effectively reverse the resistance of GSCs to TMZ and significantly enhance the cytotoxic effect of TMZ on GSCs.

To explore more effective delivery strategies, we co-loaded TMZ and Cur (4 μM) into PLGA NPs. As shown in Fig. 4E, the drug-loaded PLGA NPs (TMZ/Cur@NPs) further enhanced the inhibitory effect on GSCs, with the IC_50_ value of TMZ further reduced to 72.6 ± 10.85 μM, a 2.7-fold decrease compared to the free TMZ/Cur combination group (*p* < 0.01) and a 24-fold decrease compared to the TMZ alone group (p < 0.001). This indicated that drug loading into NPs not only retained the synergistic effect of the free drug combination but also significantly enhanced this synergistic effect, possibly due to the improved drug stability, enhanced cellular uptake, or altered drug release kinetics provided by the NPs.

To verify the effects of the drugs on GSCs from a morphological and stem cell marker expression level, we observed GSCs tumor spheroids after different drug treatments under an inverted fluorescence microscope (Fig. 4F). Bright field observations revealed that, compared to the Control group and TMZ alone group (TMZ), the tumor spheroids in TMZ/Cur combination group (TMZ/Cur) had a looser structure with more dead or detached cells. More importantly, the fluorescence intensity of CD133 labeled with Cy5.5 was significantly reduced in the TMZ/Cur group. CD133 is a key marker for maintaining the stemness of GSCs, and its downregulation intuitively reflected that the combination of TMZ and Cur can effectively inhibit the stemness of GSCs.

The results clearly demonstrate the significant resistance of GSCs to TMZ (IC_50_ > 1700 μM), which is closely related to their self-renewal and enhanced DNA repair capabilities, an important root cause of GBM recurrence. Cur alone showed strong inhibitory activity against GSCs (IC_50_ ∼ 15 μM), indicating its ability to directly target GSCs. The most critical finding is that even a low dose (IC_25_) of Cur can effectively overcome the resistance of GSCs to TMZ, reducing the IC_50_ of TMZ by nearly 9-fold. This was highly consistent with the “sensitizing tumor cells to TMZ chemotherapy” and “inhibiting GSC stemness” aspects of the “triple-action” effect of Cur proposed in the introduction. The downregulation of CD133 and the disruption of tumor spheroids structure further confirmed at the phenotypic level the inhibitory effect of the drug combination on the stemness of GSCs, supporting the network pharmacology-predicted targets (such as potentially affecting stemness maintenance through the inhibition of JAK/STAT, EGFR, and other pathways) (Neradugomma et al., 2013).

After co-loading TMZ and Cur into PLGA NPs, the cytotoxic effect on GSCs was further enhanced, achieving a 24-fold increase in efficacy. This enhancement may stem from several factors: 1) NPs protected Cur from degradation, maintaining its bioactivity; 2) NPs facilitated the co-delivery of drugs into the interior of GSCs, increasing local drug concentrations; 3) The sustained-release characteristics of NPs may prolong the duration of drug action in the GSC culture system, enhancing the synergistic effect. The most significant decrease in CD133 in the TMZ/Cur@NPs group also supports that nanoparticle delivery can more effectively inhibit stemness. These results provided important in vitro experimental evidence for the subsequent construction of a PIDDS that simultaneously delivers TMZ and Cur, and strongly suggest that this strategy has the potential to more effectively eliminate the TMZ-resistant GSC “seeds” remaining after surgery, thereby inhibiting recurrence.

The significantly enhanced cytotoxicity of TMZ/Cur@NPs compared to the free drug combination (IC_50_ reduced from 195.9 μM to 72.6 μM, Fig. 4E) underscored the critical role of the PLGA nano-delivery system beyond mere passive carrier functions. While the free drug combination established the intrinsic pharmacological synergy, the nanoparticle formulation dramatically potentiated this effect. This enhancement can be attributed to the fundamental pharmaceutical advantages offered by the NPs, which are central to the scope of this study: improved cellular uptake due to the nanoscale size, enhanced drug stability (particularly for the hydrophobic and unstable Cur), and controlled release kinetics that likely maintain an effective intracellular drug concentration for a prolonged duration. Therefore, the PLGA NPs are not inert but are active contributors to the therapeutic outcome by optimizing the biopharmaceutical properties of the drug duo.

### 3.4 Western blot and cellular metabolic level determination

#### 3.4.1 Western blot

**Fig. 5:**
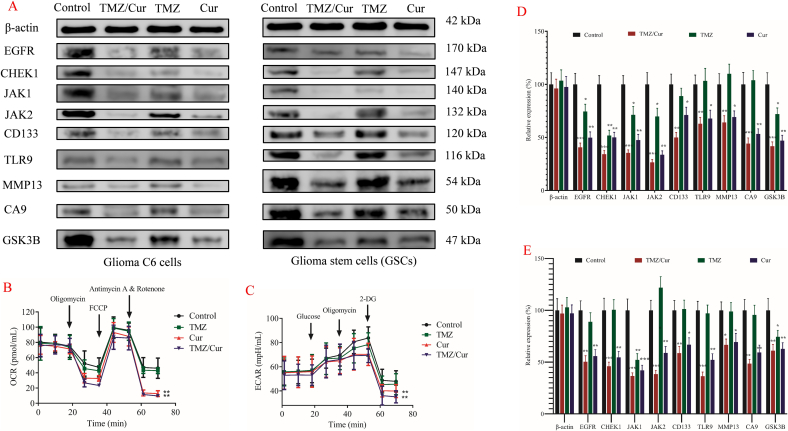
Western blot and metabolic level changes of TMZ/Cur combination in inhibiting tumor cells. A: Western blot results of TMZ/Cur combination inhibiting tumor cells. Left: Glioma C6 cells; Right: GSCs. B: Changes in mitochondrial respiration (OCR) of GSCs inhibited by TMZ/Cur combination; C: Changes in glycolysis levels (ECAR) of tumor cells inhibited by TMZ/Cur combination; D: Quantitative analysis of Western blot for C6 cells by TMZ/Cur combination; E: Quantitative analysis of Western blot for GSCs by TMZ/Cur combination.

To further investigate the effects of TMZ and Cur alone and their combination on the expression of key signaling pathway proteins in C6 cells and GSCs, we conducted Western blot analysis to detect the expression levels of the 9 key targets predicted by network pharmacology (EGFR, CHEK1, JAK1, JAK2, CD133, TLR9, MMP13, CA9, GSK3B) (Fig. 5A).

Upon quantitative analysis of the WB results, it was found that when the drugs acted on glioma cells C6 (Fig. 5D), compared to the Control group, TMZ alone significantly reduced the expression of CHEK1 protein (*p* < 0.01), decreased the expression of EGFR, JAK1, JAK2, and GSK3B (*p* < 0.05). However, there were no significant change in the expression of CD133, TLR9, MMP13, and CA9. This indicated that TMZ mainly exerted its cytotoxic effects by inhibiting the key factor CHEK1 in DNA damage repair and some growth factor/kinase signaling (EGFR/JAK). When Cur alone acted on glioma cells C6, it exhibited a broader and more potent inhibitory effect (Fig. 5D). Compared to the Control group, Cur significantly downregulated the protein expression of EGFR, CHEK1, JAK1, JAK2, GSK3B, and CA9 (*p* < 0.01), as well as CD133, TLR9, and MMP13 (*p* < 0.05). This confirmed that Cur has multi-target inhibitory properties, affecting not only proliferation/survival signaling (EGFR/JAK), DNA repair (CHEK1), and stemness (CD133), but also epigenetic/tumorigenesis (TLR9/GSK3B), invasion (MMP13), and hypoxia adaptation (CA9). Importantly, the combination of TMZ/Cur produced a strong synergistic inhibitory effect (Fig. 5D). Compared to the Control group, the expression of EGFR, CHEK1, JAK1, JAK2, GSK3B, and CA9 was extremely significantly downregulated (*p* < 0.001), and CD133, TLR9, and MMP13 were also significantly downregulated (p < 0.01). This indicated that the combination of TMZ and Cur almost covered all the detected targets, with a degree of inhibition far exceeding that of TMZ alone, confirming the multi-target synergistic mechanism predicted by network pharmacology.

Further investigation into the effects of the 2 drugs on GSCs (Fig. 5E), once again highlighted the strong resistance of GSCs to TMZ. Compared to the Control group, the TMZ alone group only observed a significant decrease in GSK3B (p < 0.05), with no significant changes in the expression of the other 8 target proteins. This was consistent with the high IC_50_ of TMZ against GSCs reported in Section 3.2. Cur alone also demonstrated a powerful multi-target regulatory ability on GSCs. Compared to the Control group, Cur alone significantly inhibited JAK1 (p < 0.001), as well as the expression of EGFR, CHEK1, JAK2, TLR9, GSK3B, CA9 (p < 0.01), and CD133, MMP13 (p < 0.05). This clearly indicated that low-dose Cur can effectively target key pathways in GSCs, including stemness maintenance (CD133/JAK1), DNA repair (CHEK1), pro-inflammatory/immune evasion (TLR9), invasion (MMP13), and hypoxia adaptation (CA9). More importantly, the combination of TMZ/Cur also showed remarkable synergistic effects in GSCs. Compared to the Control group, the combination of TMZ/Cur significantly downregulated EGFR, CHEK1, JAK1, JAK2, TLR9 (p < 0.001), CD133, GSK3B, CA9 (p < 0.01), and MMP13 (p < 0.05). Notably, targets that were ineffective with TMZ alone (such as EGFR, CHEK1, JAK1, JAK2, TLR9, CD133, MMP13) were significantly inhibited when combined with Cur, with a degree of inhibition comparable to or stronger than Cur alone (e.g., CHEK1, JAK1, JAK2, TLR9). This provided a direct molecular mechanism explanation for the ability of the combination therapy to effectively overcome GSCs resistance: Cur inhibited pathways such as JAK/STAT (stemness), TLR9 (tumor/immune suppression), and CA9 (hypoxia adaptation), while also weakening the DNA repair barrier mediated by CHEK1, thereby significantly enhancing the cytotoxic effects of TMZ on resistant GSCs (Diki et al., 2025).

The Western blot results provided strong molecular evidence supporting the predicted synergistic target pathways of TMZ and Cur by network pharmacology. In C6 cells, Cur alone demonstrated extensive target inhibition capabilities, and the combination with TMZ achieved nearly global and more potent inhibition. In the critical drug-resistant GSCs model, the results were particularly significant: 1) TMZ alone was almost ineffective, only slightly downregulating GSK3B, highlighting the resistance barrier of GSCs; 2) Cur alone efficiently inhibited multiple targets, effectively targeting stemness (CD133/JAK1/JAK2), DNA repair (CHEK1), tumor/immune evasion (TLR9), invasion (MMP13), and hypoxia adaptation (CA9) pathways; 3) the combination of TMZ/Cur achieved strong synergy, with Cur not only inhibiting key targets itself but more importantly reversing the resistance of GSCs to TMZ, allowing TMZ to effectively downregulate targets that were originally ineffective in GSCs (such as CHEK1, JAK1/2, EGFR, TLR9, etc.). This explained the molecular basis for the significant reduction in the IC_50_ of TMZ against GSCs: Cur inhibited JAK/STAT (stemness maintenance), TLR9 (pro-inflammatory/immune suppressive microenvironment), and CA9 (hypoxia adaptation) while weakening CHEK1-mediated DNA repair capabilities, thereby significantly enhancing the toxicity of TMZ against GSCs.

#### 3.4.2 Inhibition of metabolic reprogramming

Metabolic reprogramming (especially enhanced glycolysis) is a key characteristic of tumor cells (particularly GSCs) in adapting to the microenvironment, maintaining stemness, and promoting progression. We used the Seahorse XF analyzer to assess the effects of different drug treatments on the glycolytic (ECAR) and mitochondrial respiratory (OCR) functions of GSCs.

When TMZ alone was applied, there were no significant changes in the extracellular acidification rate (ECAR) or the oxygen consumption rate (OCR,) of GSCs (Figs. 5B & C). This was consistent with the Western blot results showing that TMZ alone had no significant effect on most targets (especially potential metabolism-related targets such as CA9 and GSK3B) and its relatively poor cytotoxicity. In contrast, both Cur alone and the TMZ/Cur combination group exhibited significant inhibition of GSCs' energy metabolism. Notably, the degree of metabolic inhibition in the combination group was similar to that of the Cur alone group, suggesting that the metabolic inhibition was primarily driven by Cur.

The Seahorse metabolic analysis further revealed another important mechanism of Cur (alone or in combination) in inhibiting GSCs: reprogramming energy metabolism. Cur significantly inhibited both glycolysis (ECAR↓) and mitochondrial respiration (OCR↓) in GSCs, indicating its ability to disrupt the two major energy-producing pathways that GSCs rely on (Olanlokun et al., 2024). This global metabolic suppression severely impaired GSCs' ability to survive, proliferate, maintain stemness, and adapt to stress (such as postoperative hypoxia/oxidative stress). CA9, an important pH regulator and hypoxia marker, was significantly downregulated by Cur, which may be one of the key factors leading to glycolysis inhibition (Swietach et al., 2007), as CA9 helps maintained the acidic microenvironment required for glycolysis. The metabolic inhibition, together with the aforementioned target inhibition (such as inhibiting survival/proliferation-promoting JAK-STAT and EGFR signaling), formed the solid molecular and phenotypic basis for Cur's “triple-action” effect (sensitizing TMZ, inhibiting stemness, and modulating the microenvironment), providing the core theoretical support for the subsequent design of a PIDDS based on the synergistic effects of the 2 drugs.

This section, through analysis at both the protein and functional metabolic levels, has confirmed at the molecular mechanism level that Cur overcome GSCs resistance through multi-target inhibition, thereby enhancing TMZ efficacy, inhibiting GSCs stemness, and modulating the related microenvironment. It also revealed its reprogramming effect on tumor energy metabolism, providing a key mechanistic basis for the use of TMZ/Cur co-loaded ROS-sensitive biphasic drug-release PIDDS to inhibit postoperative recurrence of GBM. It is noteworthy that the potent multi-target inhibition and metabolic reprogramming effects demonstrated by the free TMZ/Cur combination provide the mechanistic blueprint for the system's action. The primary role of the TMZ/Cur@NPs, as evidenced by the superior cytotoxicity (Fig. 4E), is to ensure that this blueprint is executed more efficiently in vivo. The NPs facilitate the co-delivery of both drugs to the same cell population (especially the hard-to-target GSCs), protect the payload from premature degradation, and provide a sustained release that could lead to more durable pathway suppression. Future studies will directly compare the pathway inhibition profiles of free drugs versus NP-encapsulated drugs to decipher any potential differences in their pharmacodynamic interactions.

### 3.5 Pharmacodynamics

**Fig. 6:**
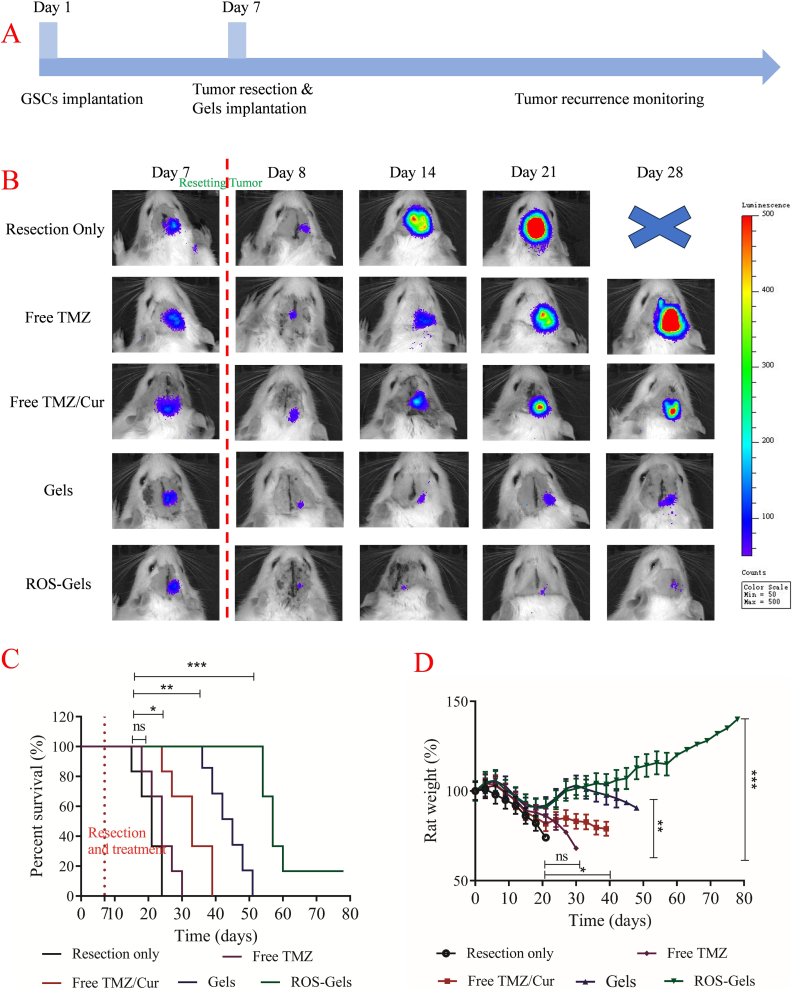
Pharmacodynamics of gel PIDDS. A: PIDDS workflow. B: Living image of rat at Day 7, Day 8, Day 14, Day 21 and Day 28 during pharmacodynamics. C: Rat survival time. D: Body weight change of rat.

The pharmacodynamics workflow of the PIDDS was shown in Fig. 6A, with living imaging performed every 7 days to monitor the glioma recurrence. Fig. 6B showed the living imaging results on Day 7 (before tumor resection), Day 8 (after tumor resection and drug implantation), Day 14, Day 21, and Day 28 to determine the recurrence status of glioma. As shown in Fig. 6B, on Day 7 (preoperative), all groups exhibited similar and strong bioluminescent signals, confirming the successful construction of the intracranial glioma model by GSCs. On Day 8 (24 h postoperative), the tumor signals in all groups were significantly reduced (resection rate > 90 %), but residual weak signals (blue fluorescence) remained, consistent with the infiltrative growth characteristics of GBM, indicating the presence of residual tumor cells. From Day 14 to Day 28: the Resection only group showed a sharp increase in signal, indicating rapid tumor recurrence; the Free TMZ group had a recurrence rate similar to the control group (Resection only); while the Free TMZ/Cur group showed delayed recurrence. Notably, the drug-loaded gel groups (especially ROS-Gels) maintained the lowest signal intensity throughout the period, showing only weak fluorescence by Day 28.

The survival curves (Fig. 6C) revealed that the median survival time in the Resection only group was only 19 days, confirming the clinical dilemma of extremely rapid recurrence after GBM surgery. The median survival time in the Free TMZ group was 22 days (*p* > 0.05 vs. the control group), highlighting the difficulty of TMZ effectively inhibiting residual brain tumor especially GSCs when administered systemically. The median survival time in the Free TMZ/Cur group was extended to 33 days (*p* < 0.05 vs. the control group), confirming the efficacy of Cur in synergistically sensitizing TMZ, although still limited by rapid drug clearance. The Gels group (non-ROS-sensitive gel PIDDS) achieved a median survival time of 43 days (*p* < 0.01 vs. the control group), demonstrating the advantages of local drug delivery. The ROS-Gels group (ROS-sensitive gel PIDDS) achieved a breakthrough: the median survival time was significantly extended to 57 days (p < 0.05 vs. the Gels group), with 1 of the 6 rats surviving long-term (>60 days), a threefold increase in survival compared to the control group.

In the rat body weight assessment shown in Fig. 6D, the Resection only group, Free TMZ group, and Free TMZ/Cur group experienced continuous weight loss after surgery, indicating tumor progression and chemotherapeutic toxicity. The weight loss in the Gels and ROS-Gels groups was significantly reduced (103 % and 101 % of the initial weight at Day 28, respectively), with the ROS-Gels group maintaining the best condition (*p* < 0.001 vs. the free combination group), proving that local drug delivery by PIDDS effectively reduced systemic toxicity.

In this study, the significant survival advantage of the ROS-Gels group (57 days vs. 43 days in the Gels group) was attributed to its dual design of biphasic drug release and the “triple-action” effect of Cur. During the rapid release phase of ROS-Gels, free TMZ/Cur is immediately released after surgery to synergistically eliminate residual cells. During the sustained release phase, the ROS-sensitive gel responds to the postoperative tumor microenvironment, selectively releasing PLGA NPs co-loaded with TMZ/Cur, thereby achieving long-term inhibition of GSCs stemness and microenvironment modulation to prevent recurrence. This design simultaneously addresses 3 major challenges: the “seed” (TMZ/Cur synergistically inhibiting GSCs), the “soil” (modulating the postoperative recurrence microenvironment), and overcoming the drug release defects of the current PIDDS system (extending the drug release cycle to 30 days). This ROS-Gels, through its “rapid clearance + long-term regulation” biphasic drug release, combined with Cur's “triple-action” effect (sensitizing chemotherapy, eradicating GSCs, and modulating the microenvironment), provides an effective solution to overcome postoperative recurrence of GBM.

### 3.6 HE staining and TUNEL staining

**Fig. 7:**
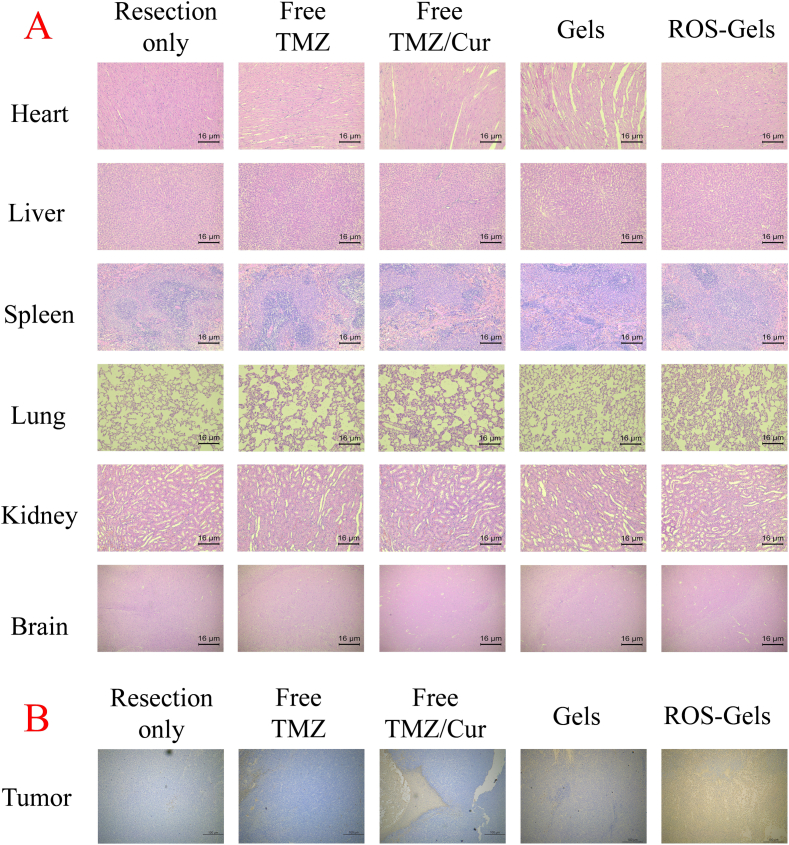
HE staining and TUNEL staining. A: HE staining. B: TUNEL staining.

To evaluate the systemic safety of different therapeutic strategies, H&E staining was performed on heart, liver, spleen, lung, kidney, and brain tissues (Fig. 7A). The results showed that in the free drug groups (Free TMZ group and Free TMZ/Cur group), the liver exhibited increased hepatic congestion, and the lung tissue showed thickened alveolar walls, indicating hepatic and pulmonary toxicity due to systemic drug exposure. In the two PIDDS groups (Gels group and ROS-Gels group), the structure of each organ was intact, with no significant pathological change and consistent with the morphology of normal tissues, confirming that local drug delivery effectively avoided systemic toxicity.

On postoperative Day 14, TUNEL staining was performed on recurrent tumor tissues (Fig. 7B) to quantify the proportion of apoptotic cells. In the Resection only group, very few apoptotic cells were observed, with dense proliferation of recurrent tumor cells. In the free drug groups (Free TMZ group and Free TMZ/Cur group), the proportion of apoptotic cells increased slightly, but a large number of tumor cells still survived, with a clear trend of recurrence. In the PIDDS groups, apoptotic signals were significantly enhanced, especially in the ROS-Gels group (brown signal). Combined with the living imaging results from the pharmacodynamics section, this indicated that the ROS-Gels group had the smallest recurrent tumor volume, and the in situ release of drugs in this group efficiently induced tumor cell apoptosis.

### 3.7 Advantages, limitations and future perspectives

In summary (Table 1), the key values of our work could be: 1) Biphasic drug release: combines rapid initial release with sustained NP-based release, addressing the shortcoming of rapid drug clearance in existing systems. 2) Curcumin's triple action: first system to leverage Cur's synergistic sensitization, stemness inhibition, and microenvironment reprogramming in a single formulation. 3) ROS-Responsiveness: enables targeted drug release in the postoperative TME, enhancing efficacy and reducing off-target effects. 4) Comprehensive mechanistic validation: supported by network pharmacology, proteomics, metabolomics, and in vivo imaging, providing a multi-level understanding of the anti-recurrence mechanism.

**Table 1:** Comparative summary of postoperative in situ drug delivery systems for glioma treatment.

Feature / Metric	Gliadel® wafer (current clinical Standard) (Attenello et al., 2008)	Other reported hydrogel systems (Qiao et al., 2022)	Our study (ROS/Thermo-sensitive PIDDS)
Drug release duration	3–4 days	Typically <14 days	> 30 days (sustained release)
Cumulative release rate	∼70 % (limited by sink effect)	∼60–80 %	> 88 % (both TMZ and Cur)
Sensitivity to TME	No	Limited (pH/enzyme)	Yes (ROS + thermo)
Targets GSCs	No	Rarely	Yes (via Cur “three-in-one” effect)
IC₅₀ reduction for TMZ	Not applicable	Not reported	9-fold (free) → 24-fold (NPs-loaded)
Median survival (rat)	∼28–35 days (literature)	∼40–45 days	57 days (3× control)
Systemic toxicity	High (systemic exposure)	Moderate	Low (localized delivery)
Mechanistic insight	Limited	Partial	Comprehensive (multi-omics + functional assays)
Microenvironment modulation	No	Limited	Yes (hypoxia, immune, metabolism)

Despite the promising results, this study has limitations that must be addressed for clinical translation. A deliberate focus of the mechanistic studies (Sections 3.3.1 and 3.3.2) was to first establish the intrinsic synergistic pharmacology between TMZ and Cur, decoupled from delivery effects. However, we fully acknowledge that evaluating the biological performance of the delivery system itself is paramount in pharmaceutical sciences. Our data already provide strong indirect evidence of the PLGA NPs' vital role, as the TMZ/Cur@NPs formulation showed a 2.7-fold increase in potency over the free drug combination (Fig. 4E). A key limitation of this study is the absence of a direct, head-to-head comparison of the mechanistic profiles (e.g., Western blot, metabolic analysis) between the free drug combination and the NP-encapsulated drugs. Therefore, a primary and essential direction for our immediate future research is to conduct such a comparative analysis. This will determine whether the enhanced efficacy stems solely from superior pharmacokinetics or also from altered pharmacodynamics, such as sustained intracellular signaling pathway inhibition or differential subcellular trafficking enabled by the nanoparticle carrier. Furthermore, the primary constraint is the use of a rodent model, which does not fully recapitulate the scale of a human resection cavity or the complexity of the human immune microenvironment.

Future studies will focus on comparing the mechanisms of free drugs versus NPs to determine if efficacy improvements are due to pharmacokinetic enhancement versus distinct pharmacodynamic effects. Validation in large animal models that better simulate human surgical and immune environments will be pursued. Additionally, scalable GMP-compliant production and long-term safety studies of the gel will be essential for clinical translation.

## 4 Conclusion

This study successfully constructed a ROS-sensitive PIDDS, which integrates the “triple-action” effect of Cur with a biphasic drug-release design to break through the postoperative treatment bottleneck of GBM. The first aspect is overcoming drug resistance and eliminating GSCs. Cur works in synergy with TMZ to downregulate the CHEK1/JAK-STAT pathway, reversing GSCs resistance (IC_50_ reduced from 1765 μM to 72.6 μM) and depleting stem cells. The second aspect is precise microenvironment modulation. ROS-Gels respond to the postoperative tumor microenvironment, continuously releasing drugs to inhibit TLR9/CA9 and block the tumor-hypoxia vicious cycle. The third aspect is long-term tumor control and safety. The biphasic drug release covers the critical 30-day recurrence period, extending the median survival time to 57 days (3 times that of the surgery-only group). H&E staining confirmed no significant toxicity, addressing the issues of Gliadel®’s short release cycle and systemic toxicity. This strategy provides a new therapeutic model for clinically inhibiting GBM recurrence, which is highly efficient, safe, and microenvironment-responsive.

## Author contribution declaration

Qiong Liang and Jiangjian Liu contributed equally to this work and are co-first authors. Qiong Liang was responsible for the conceptualization of the study, data collection, and initial drafting of the manuscript. Jiangjian Liu contributed to the experimental design, data analysis, and interpretation of the results. Sunhui Chen, the corresponding author, provided overall supervision, guided the research direction, and critically revised the manuscript for important intellectual content. Yanhang Zhuo contributed to the literature review, assisted in data analysis, and provided technical support throughout the study. All authors reviewed and approved the final version of the manuscript.

## Funding

This work was supported by the Fujian provincial health technology project (No. 2022GGA006), and Joint Funds for the innovation of science and Technology of Fujian Province (No. 2023Y9274).

## CRediT authorship contribution statement

**Qiong Liang:** Writing – original draft, Visualization, Validation, Supervision, Software, Resources, Project administration, Methodology, Investigation, Funding acquisition, Data curation, Conceptualization. **Jiangjian Liu:** Writing – original draft, Visualization, Validation, Supervision, Software, Resources, Project administration, Methodology, Investigation, Formal analysis, Data curation, Conceptualization. **Yanhang Zhuo:** Software, Resources, Project administration, Methodology, Conceptualization. **Sunhui Chen:** Writing – review & editing, Writing – original draft, Visualization, Validation, Supervision, Software, Resources, Project administration, Methodology, Investigation, Funding acquisition, Formal analysis, Data curation, Conceptualization.

## Declaration of competing interest

The authors declare that they have no known competing financial interests or personal relationships that could have appeared to influence the work reported in this paper.

## Data Availability

Data will be made available on request.

## Appendix A Supplementary data

Supplementary material Image 1
