# Supplementary material for: A postoperative in situ drug delivery system based on biphasic drug-release and “Three-in-One” Effect of curcumin to inhibit the recurrence of glioma
Source: Int J Pharm X. 2025 Oct 12;10:100418. doi: 10.1016/j.ijpx.2025.100418 (PMC12552640; doi:10.1016/j.ijpx.2025.100418)
Supplement: Supplementary file 1 — Supplementary material [file mmc1.docx]

**Supplemental Table 1.** Characteristics of PLGA NPs

| Characteristics | Parameters |
| --- | --- |
| Size (nm) | 122.4 ± 10.5 |
| PDI | 0.098 ±0.025 |
| Zeta potential (mV) | -23.8 ± 2.6 |
| Cur EE (%) | 79.8 ± 1.8 |
| TMZ EE (%) | 66.5 ± 3.3 |
| Cur DL (%) | 4.9 ± 0.4 |
| TMZ DL (%) | 10.7 ± 1.1 |
